# Recent Progress in Distiller’s Grains: Chemical Compositions and Biological Activities

**DOI:** 10.3390/molecules28227492

**Published:** 2023-11-09

**Authors:** Ran He, Yubo Yang, Yongsu Li, Minghua Yang, Lingyi Kong, Fan Yang

**Affiliations:** 1 Jiangsu Key Laboratory of Bioactive Natural Product Research and State Key Laboratory of Natural Medicines, School of Traditional Chinese Pharmacy, China Pharmaceutical University, Nanjing 210009, China; hr06072022@163.com (R.H.); cpu_lykong@126.com (L.K.); 2 Kweichow Moutai Co., Ltd., Zunyi 564501, China

**Keywords:** distiller’s grains, development, utilization, chemical compositions, bioactivities

## Abstract

Distiller’s grains (DGs) are solid mixtures that remain after the production of alcoholic beverages. A large amount of DGs is produced each year during the brewing process. Currently, they are mostly used as a feedstock or substrate in the feed industry. However, the lack of a comprehensive understanding of the chemical composition of DGs is a major constraint on their further development and application for high-value-added usages. Some studies were published on the bioactive constituents of DGs in several different types of journals. Data were therefore collated to provide a comprehensive overview of these natural products. DGs are rich in phenols, phytosterols, and fatty acids, in addition to general lipid and protein constituents. These compounds and their related extracts possess diverse biological activities, including antioxidant, anti-inflammatory, and anti-hyperglycaemic effects. We hope that this review will provide research incentives for the further development and utilisation of DGs to develop high-value-added products.

## 1. Introduction

There is a wide variety of alcoholic beverages worldwide. A staggering amount of distiller’s grains (DGs), a major by-product of the brewing industry, is produced by brewing enterprises each year. According to incomplete statistics, this amount exceeds 100 million tons annually [1]. The problem caused by such a large amount of DGs is how to handle and use them. DGs are rich in moisture and nutritional ingredients, which makes them susceptible to spoilage and deterioration. If they cannot be processed effectively in a timely and effective manner, they will have a substantial impact on the ecological environment because they are prone to mould, owing to their high moisture content. Currently, DGs are mainly used to produce aquaculture feed and biogas for secondary energy utilisation and as a raw material for producing fertilisers for the agricultural industry [2]. In general, these are traditional methods for recycling DGs; however, they incur high economic costs and do not efficiently take advantage of DGs with respect to the economic value of the derived products.

In addition to nutritional components, DGs contain bioactive natural products. In the past decades, compounds and extracts from DGs were reported to exert diverse bioactivities, suggesting that they could be good sources for developing high-value-added derivatives. However, they have not yet received much attention. Most previous studies focused on the use of DGs as feed and fertilisers; thus, studies focused on the analysis of sugars, fatty acids, and proteins in DGs [3]. Notably, active compounds may be embedded in complex DG constituents and are, therefore, technically difficult to extract or purify. Another possible reason is that these bioactivity-related reports are scattered among journals in different fields. The potential applications of DGs have not yet been systematically determined. Currently, there are no reviews that comprehensively summarise the bioactive constituents of DGs. Liu et al. reviewed the chemical composition of DGs in 2011 [4] and focused on changes in nutrient levels during DG processing. To the best of our knowledge, the first review on bioactive natural products from DGs was published in 2019 and only summarises the antioxidant phenolic compounds in wine lees [5]. Therefore, an in-depth review of the literature covering the bioactive chemical composition and possible biological activities of different DG extracts was conducted. This review provides a more comprehensive understanding of these bioactive components, which is expected to facilitate the comprehensive utilisation of DGs based on their biological activities.

## 2. Research Progress on the Chemical Constituents of DGs

### 2.1. Volatile Constituents

DGs contain various volatile constituents that produce diverse aromas. Li et al. [6] used gas chromatography–mass spectrometry (GC–MS) to analyse the aroma and flavouring substances in a DG extract from a Moutai-flavoured liquor. The identified compounds included alcohols, acids, esters, hydroxyl compounds, aldehydes, nitrogen-containing compounds, and heterocyclic compounds. Similarly, Zhang et al. [7] first enriched the volatile constituents of Moutai-flavoured DGs by steam distillation and then analysed them using GC–MS. A total of 32 species were identified, including furfural, ethyl linoleate, ethyl palmitate, ethyl oleate, 5-methyl-2-furaldehyde, phenethyl alcohol, pyrrole-2-carbaldehyde, benzaldehyde, phenylacetaldehyde, and 2-acetylfurane. Huang et al. [8] used *Pichia kudriavzevii* and *Irpex cacteus* to treat DGs from a sauce-flavoured liquor and analysed the differences in volatile composition before and after fermentation. The results showed that 54, 44, and 37 volatile constituents were detected in the original DGs, *P. kudriavzevii*-fermented DGs (DG-P), and *I. cacteus*-fermented DGs (DG-I), respectively. The relative content gradually decreased. The volatile constituents of the original DGs and DG-P were esters (57.85% and 47.25%, respectively) and alcohols (27.67% and 30.92%, respectively), while the main volatile constituents of DG-I were alkenes (48.76%). Lei et al. [9] used a combination of headspace solid-phase microextraction (HS-SPME) and GC–MS to analyse the spatial distribution of the aroma constituents of DGs. The main aromatic constituents of DGs appeared to be esters and acids, including hexanoic acid, butyric acid, ethyl hexanoate, ethyl butyrate, hexyl hexanoate, and butyl hexanoate. Furthermore, Jiang et al. [10] used HS-SPME and GC–MS to analyse the aromatic components of an aqueous DG extract. Twenty aroma constituents were identified, including esters, acids, alcohols, and aldehydes, of which esters accounted for more than half. Ye et al. [11] identified 31 volatile constituents in a recycled leachate of fresh DGs by GC–MS analysis, including 8 lipids, 10 acids, 9 alcohols, and 4 other substances. Moreover, Wang et al. [12] used physical and chemical detection and GC–MS to analyse a recycled leachate of fresh DGs in each phase of liquor production and then combined the results with the aroma activity value to analyse the flavour constituents of DGs. They identified 39 flavour substances in DGs, mainly hexanoic acid ethyl ester, octanoic acid ethyl ester, linoleic acid ethyl ester, hexanoic acid, butyric acid, valeric acid, n-hexanol, furfuryl alcohol, and 3-hydroxy-2-butanone.

Roth et al. [13] analysed the flavour compounds in brewer grains (BGs). They used headspace solid-phase extraction, solvent-assisted extraction, distillation, gas chromatography, olfactory methods, and spectroscopy for their identification. A total of 42 odour-active compounds were identified, including 3-methylbutyric acid, dimethyl trisulfide, 4-hydroxy-2-furanone, 2,6-ethyl-3-hydroxy-4-methyl-2-furanone, and 2-ethyl-3,5-methylpyrazine phenethyl alcohol.

In conclusion, the volatile constituents of DGs are mostly higher alcohols, esters, acids, and other substances with high boiling points. This is because many molecules with low molecular weights and boiling points evaporate during the production process of alcoholic beverages. Ethyl lactate and ethyl caproate are common and representative volatile components of different DGs, in addition to edible flavours widely used in the industry. Thus, the volatile constituents of DGs can potentially be developed into essences, for which a refining technique is required.

### 2.2. Fatty Acids

Gómez et al. [14] used solvent extraction to extract the lipid constituents of Spanish sherry and then analysed the extract constituents using gas chromatography (GC). Thirteen fatty acids were identified in the extract: capric acid, lauric acid, myristic acid, palmitic acid, palmitoleic acid, margaric acid, stearic acid, oleic acid, linoleic acid, linolenic acid, arachidonic acid, erucic acid, and lignoceric acid. The highest content was found for palmitic acid (33.29%), followed by linoleic acid (21.26%). Farcas et al. [15] analysed the fatty acid composition of BG samples using gas chromatography GC–MS. A total of 26 fatty acids were identified in the samples, with the most abundant being linoleic, palmitic, and oleic acids. The samples also contained linolenic and stearic acids and the less abundant myristic, vaccenic, arahidic, 11-eicosenoic, behenic, and lignoceric acids. This is similar to the composition of the lipid constituents of barley, which is the main BG component.

Wang et al. [16] identified polyalcohols in sorghum DGs. They used n-hexane or thermal ethanol to extract the lipid constituents from sorghum DGs and analysed them using high-performance liquid chromatography (HPLC). The main constituents were found to be polyalkanes, accounting for 37–44% of the components, followed by aldehydes (44–55%) and acid constituents (4–5%).

Wang et al. [17] used a supercritical fluid to extract the lipid constituents from sorghum DGs. By comparing the mass ratios of different carbon dioxide and dry grains, the effect of extraction pressure, extraction temperature, and time on the yield and composition of the extracted lipids was determined to obtain lipid extracts under optimal extraction conditions. The maximum lipid yield was 50 g/kg. The specific constituents of the extract were analysed and found to be mainly tocopherols, phytosterols, polyalkanols, and free fatty acids. The content of free fatty acids was 155.3 mg/g, and palmitic acid, oleic acid, and linoleic acid were the three main free fatty acids extracted, accounting for approximately 94% of all free fatty acids.

In summary, DGs mainly contain 16- and 18-carbon fatty acids. The most abundant acids are palmitic, linoleic, and oleic acids. Palmitic acid is a widely used industrial chemical material. For example, it is used as a surfactant and oil component in cosmetics. Linoleic and oleic acids are also used in the industry to produce surfactants and skin conditioners. Additionally, they are the most nutritionally valuable, unsaturated fatty acids and can prevent atherosclerosis and lower the cholesterol levels. Therefore, rich fatty acids should be considered important constituents when synthetically utilising DGs for the production of high-value-added products.

### 2.3. Protein and Amino Acid Constituents

DGs mainly consist of globulin, gluten, alcohol-soluble proteins, and glutenin [18]. The nutritional value of the proteins contained in DGs depends on the type and quantity of their amino acids, particularly the essential ones. Cromwell et al. [19] reported that in nine different DG samples from beverage or fuel alcohol production systems, the content of lysine was 0.48–0.97%, the content of methionine was 0.49–0.61%, the content of threonine was 0.99–1.28%, and the content of tryptophan was 0.18–0.25%. Spiehs et al. [20] analysed 119 dry DG samples and found that the lysine content was approximately 0.85% on a dry matter basis. They found that BGs were a good source of essential and non-essential amino acids. The essential amino acids in BGs are methionine, phenylalanine, tryptophan, histamine, and lysine, whereas the nonessential amino acids are serine, alanine, glycine, and proline [21]. Distiller’s dried grains with solubles (DDGS) are the major and mature products of DGs and have long been marketed as animal feed for livestock. Proteins, along with amino acids, are key nutrients for fodder and have attracted much attention in studies on the production and analysis of DDGS. There are several reviews on protein value in animal nutrition and the variations in proteins depending on the material source and the processing steps [22]. These previously described findings are not reported here.

### 2.4. Bioactive Peptides

Bioactive peptides include a wide range of different peptides, from dipeptides to peptides with complex linear and cyclic structures composed of 20 natural amino acids in various amounts and arrangements. These compounds are multifunctional, protein-derived compounds. Bioactive peptides have various metabolic and physiological functions in humans. They are easily digested and absorbed and have various effects, such as promoting immunity, regulating hormones, exerting antibacterial and antiviral activities, lowering blood pressure, and reducing blood lipids. The study of bioactive peptides is currently the most popular research topic in the international food industry; these peptides are functional factors with great development prospects [23].

The separation of bioactive peptides from DGs is currently a research hotspot. Most bioactive peptides isolated from DGs exhibit a certain degree of biological activity [24], providing new research directions for the utilisation and development of DGs. Jiang et al. [25] successfully extracted bioactive peptides from DGs using ultrasonic extraction and combined these results with those form various column chromatography analyses to identify bioactive peptides from DGs. Two tripeptides were obtained, VNP and YGD, and a range of antioxidant activity tests were carried out on them.

Garzon et al. [26] purified peptides from BGs using anion-exchange, gel filtration, and reversed-phase HPLC. The peptides were structurally identified using matrix-assisted laser desorption ionisation time-of-flight mass spectrometry (MALDI-TOF-MS), and their amino acid sequences were WNIHMEHQDLTTME, DFGIASF, and LAAVEALSTNG. Dong et al. [27] used the static adsorption of macroporous resins to separate water-soluble peptides from DGs. Four angiotensin-converting enzyme (ACE) inhibitory peptides were manually identified using ultra-high-performance liquid chromatography–tandem mass spectrometry (UPLC-Q-TOF–MS), i.e., PR, DR, LP, and NGGPPT. Triple-quadrupole mass spectrometry (ESI-QQQ–MS) in multiple reaction monitoring (MRM) was employed to quantify seven peptides, with PA being the most abundant.

Liao et al. [28] concentrated the leachate obtained during the fermentation of Chinese spirits, extracted it with ethanol, and used various column chromatography techniques to separate and purify dipeptides and cyclic dipeptides. The structures of the obtained 32 dipeptides and cyclic dipeptides in water were VA, LG, PV, LA, VY, PA, LF, PL, VL, PF, VF, LL, cyclo-PA, cyclo-EL, cyclo-EF, cyclo-PL, cyclo-IL, cyclo-PF, cyclo-AF, cyclo-PP, cyclo-AA, cyclo-LA, cyclo-AA, cyclo-AL, cyclo-FF, cyclo-LF, cyclo-PA, cyclo-PE, cyclo-PY, cyclo-PT, cyclo-FA, and cyclo-LL.

Garzón et al. [29] used Sephadex G-25 molecular-exclusion columns to separate peptides from spent sorghum grains and assayed the antioxidant, antidiabetic, and antimicrobial activities of various components. The most active components were evaluated using LC-ESI-Q-TOF tandem mass spectrometry, and six new peptides with high biological activity were identified. The amino acid sequences of these peptides were AGLPTEEKPPLL, QADPKTFYGLM, GPPKVAPGKDISASFGGEWL, GGAAGGR, PPPGSKSYGT, and AAGGAAF.

### 2.5. Phytosterols

**Figure 1:**
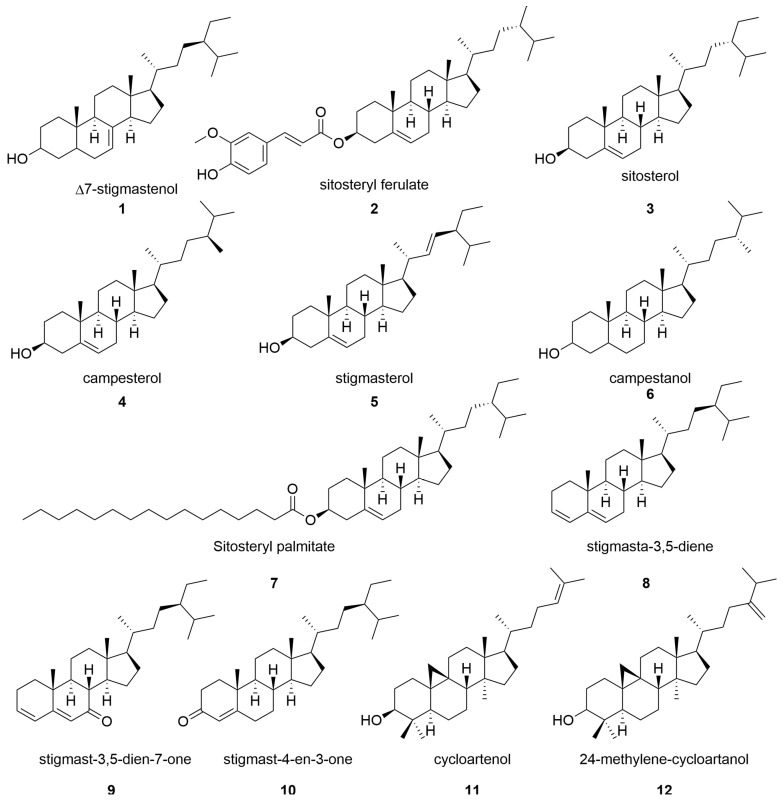
Chemical structures of phytosterols in distiller’s grains.

Phytosterols are a common class of beneficial nutrients in plants [30] and are thus potential bioactive constituents in DGs. Figure 1 shows the phytosterols reported in DGs. Winkler et al. [31] obtained an oily extract from dried DGs by molecular distillation. An HPLC analysis revealed phytosterols, such as ∆7-stigmastenol (**1**), and ferulate phytosterol esters, such as sitosteryl ferulate (**2**), in that extract. Río et al. [32] used GC–MS to identify sterols in BGs, which were mainly sterol esters, steroid ketones, sterol glycosides, free steroids, and steroid hydrocarbons.

The most abundant free sterol in BGs is sitosterol (**3**), while the lowest amounts are found for campesterol (**4**), stigmasterol (**5**), and campestanol (**6**). Sitosteryl palmitate (**7**) is the predominant sterol ester. Steroid ketones consist mainly of stigmasta-3,5-diene (**8**), stigmast-3,5-dien-7-one (**9**), and stigmast-4-en-3-one (**10**). Winkler et al. [33] used n-hexane to extract the lipid constituents of sorghum DGs and then extracted tocopherols and phytosterols. The major phytosterols were cycloartenol (**11**) and 24-methylene-cycloartanol (**12**). Leguizamón et al. [34] used reflux and Soxtec extraction to isolate phytosterols from sorghum DGs and showed that the Soxtec method could extract more phytosterols than the reflux method. Campesterol (**4**) and stigmasterol (**5**) were detected after Soxtec extraction.

The main plant sterols found in DGs are free sterols, such as sitosterol, campesterol, and stigmasterol, with sitosterol being the major component. It is worth noting that esterified sterols found in grains, such as oryzanol, are rarely reported in DGs, although they are important bioactive natural products that have health benefits. Free sterols, such as sitosterol, are anti-inflammatory and antioxidant molecules, also beneficial in reducing total cholesterol in the blood. Therefore, phytosterols are important constituents of DGs.

### 2.6. Polyphenols

**Figure 2:**
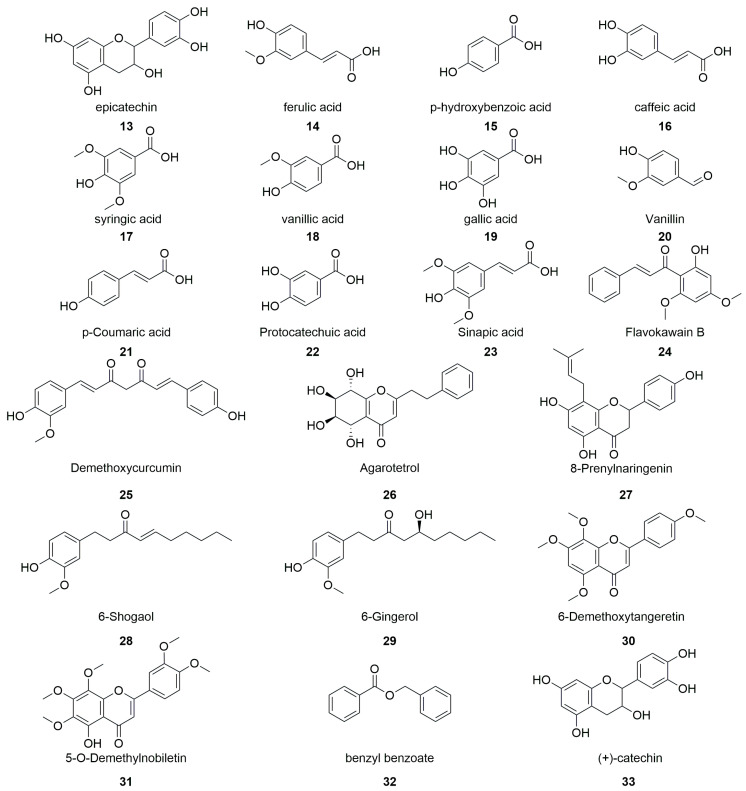
Chemical structures of polyphenols in distiller’s grains.

Natural polyphenols have received much attention because they show diverse biological activities, such as antioxidant, anti-aging, anti-radiation, antibacterial activities, and can lower blood pressure and the levels of blood lipids [35]. Polyphenols are present in a relatively high concentration in DGs, which makes them valuable constituents for further development. The polyphenols in DGs are mainly flavonoids and organic acids (Figure 2).

Wang et al. [36] used a macroporous resin to purify polyphenols from a DG extract and further analysed the polyphenol constituents using ultra-high-performance liquid chromatography–tandem mass spectrometry (UPLC-MS/MS). Epicatechin (**13**) was the main polyphenol identified, followed by ferulic acid (**14**), p-hydroxybenzoic acid (**15**), caffeic acid (**16**), and syringic acid (**17**). Additionally, vanillic acid (**18**) and gallic acid (**19**) were detected at lower concentrations.

Bonifácio-Lopes et al. [37] extracted bioactive constituents from BGs using different concentrations of ethanol and found that the polyphenol constituents had antioxidant and antibacterial effects. The polyphenols were analysed and identified using HPLC and were found to be mainly vanillin (**20**), p-coumaric acid (**21**), and protocatechuic acid (**22**). Yang et al. [38] purified the chemical constituents of DGs using a Sephadex LH-20 gel and obtained a flavonoid fraction with a total flavonoid content of 79.4%. Twenty-four compounds were successfully identified by further analysis using high-performance liquid chromatography–high-resolution mass spectrometry (HPLC–HRMS). The identified polyphenols included sinapic acid (**23**), flavokawain B (**24**), demethoxycurcumin (**25**), agarotetrol (**26**), 8-prenylnaringenin (**27**), 6-shogaol (**28**), 6-gingerol (**29**), 6-demethoxytangeretin (**30**), 5-O-demethylnobiletin (**31**), benzyl benzoate (**32**), and (+)-catechin (**33**).

Flavonoids, phenolic acids, and phenylpropionic acids appeared to be the main phenols in DGs, accounting for approximately 80% of the isolated and characterised polyphenols. Epicatechin, vanillic acid, and ferulic acid are representative compounds with antioxidant and free-radical-scavenging effects. They are also used as food preservatives and cosmetic additives. Thus, the polyphenols in DGs have utilisation potential, especially considering that their chemical synthesis has a low conversion rate, high cost, and is time-consuming.

### 2.7. Other Constituents

**Figure 3:**
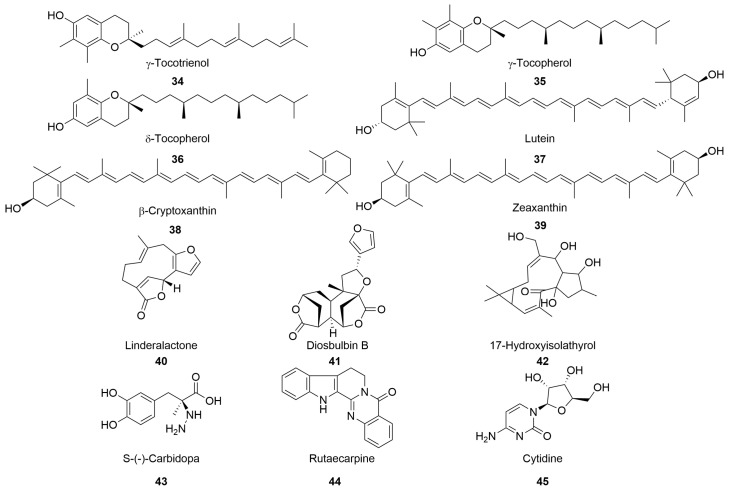
Chemical structures of other compounds in distiller’s grains.

Besides the major types of chemical constituents found in DGs detailed above, other components are reported in the relevant literature (Figure 3). Tocopherols containing γ-tocotrienol (**34**), γ-tocopherol (**35**), and small quantities of δ-tocopherol (**36**) were identified and quantified in distiller’s dried grains (DDGs) using HPLC and fluorescence detection [33].

DDG oil is a good source of carotenoids, particularly lutein (**37**), β-cryptoxanthin (**38**), and zeaxanthin (**39**) [33]. Yang et al. [38] used LC–HRMS to analyse the chemical constituents of DGs and obtained a terpene fraction, containing linderalactone (**40**), diosbulbin B (**41**), and 17-hydroxyisolathyrol (**42**). Additionally, several alkaloids were reported in DG, including s-(-)-carbidopa (**43**), rutaecarpine (**44**), and cytidine (**45**).

Dietary fibre, which is the “seventh essential nutrient for the human body”, is abundant in DGs. Although dietary fibres from different plant sources contain structurally diverse polysaccharides, they are normally classified into water-soluble and water-insoluble types according to their solubility [39]. To the best of our knowledge, there are currently no reports describing the differences between dietary fibres from DGs and those from their source plants. For more information, please refer to reviews on dietary fibres in plants [40,41].

## 3. Research Progress on the Biological Activity of DGs

### 3.1. Antioxidant Activity

Excess free radical production is associated with cancer [42], ageing [43], and many other diseases [44]. Antioxidation can, at least partly, overcome this harm. The antioxidant potential is listed as one of the main properties of DGs. Antioxidant research related to DGs has focused on phenolic extracts. In addition, some amino acids [45] and polysaccharides [46] obtained from DGs have shown antioxidant effects. Since the antioxidant compounds in wine lees were well reviewed in 2019 [5], Table 1 summarises studies on antioxidants from other DGs. Most of these studies used the 1,1-diphenyl-2-picrylhydrazyl (DPPH) and 3-ethylbenzothiazoline-6-sulfonic acid (ABTS) methods. Researchers tended to conduct antioxidant studies on brewer’s spent grain (BSG), which may be related to the ingredients used in brewing beer. Barley and wheat are the primary fermentation materials used in beer production. They are rich sources of polyphenol compounds, which are well known for their antioxidant activities. Similarly, phenolic constituents from other types of DGs also showed antioxidant activity. Naziri et al. [47] used supercritical carbon dioxide extraction to isolate a high-value chemical compound from wine lees, squalene, a natural antioxidant, which reminds us not to focus only on phenolic constituents to identify antioxidants.

### 3.2. Anti-Inflammatory Activity

Murakamia et al. [48] used the murine macrophage cell line RAW264.7 to investigate the anti-inflammatory effects of ferulic acid isolated from rice bran pitch and its novel synthetic analogue. The protein expression of COX-2 and NO synthase was decreased by these compounds, and inhibition of TNF-*α* production was also observed. Lin et al. [49] used alkaline extraction combined with ultrasonic treatment to extract proteins from Baijiu DGs and then used composite proteinase hydrolysis for 8 h to obtain peptide fractions (VPH-1, -2, -3). VPH-3 showed lower EC_50_ values in relation to reactive oxygen species (ROS) and reactive nitrogen species (RNS) radicals than VPH-1 and VPH-2. VPH-3 also substantially inhibited the production of NO and pro-inflammatory cytokines in LPS-stimulated RAW264.7 macrophages. Crowley et al. [50] reported the anti-inflammatory activity of simulated gastrointestinal digestion (SGID) products from a BSG protein hydrolysate.

**Table 1:** Studies on the antioxidant activity of different distiller’s grains (DGs).

DG Type	Constituents	Test Methods	Inhibitory Concentration	Ref.
Baijiu DG	Bioactive peptide VNP	DPPH	0.10 μmol TE/μmol	[25]
	Bioactive peptide YGD	ABTS	0.95 μmol TE/μmol	
Baijiu DG	Polysaccharides	DPPH	IC_50_ of extruded sample: 9.8 mg/mL IC_50_ of unextruded sample: 11.12 mg/mL	[46]
		ABTS	IC_50_ of extruded sample: 51.39 mg/mL IC_50_ of unextruded sample: 156.21 mg/mL	
		•OH radical scavenging	IC_50_ of extruded sample: 0.16 mg/mL IC_50_ of unextruded sample: 0.32 mg/mL	
Rice wine lees	Polyphenols	DPPH	86% at 1.4 mg/mL (soxhlet 95% ethanol extract)	[51]
		Ferrous chelating ability	91% at 1 mg/mL (soxhlet 95% ethanol extract)	
		Reducing ability	1.877 OD at 1.25 mg/mL (soxhlet 95% ethanol extract)	
BSG	Phenols	DPPH	1.28 mg TE/g of 60% (*v*/*v*) acetone extract 0.85 mg TE/g of 60% (*v*/*v*) ethanol extract	[52]
BSG	Protein hydrolysate	SOD	0.5 U/mg protein BSG against DNA damage in U937 cells under oxidative stress conditions	[53]
BSG	Phenolic acids	ABTS	0.77 mg ascorbic acid/g BSG (60% ethanol extract)	[37]
		ORAC	24.80 mg TE/g BSG (80% ethanol extract)	
Sorghum spent grains	Protein hydrolysate	ABTS	15.49% at 0.5 mg protein/mL	[26]
DDGS	Phenols	DPPH	1.49~6.53 μmol TE/g	[54]
Liquor DG	Phenols	DPPH	IC_50_ of 60% ethanol extract: 33.3 µg/mL	[55]
		ABTS	IC_50_ of 60% ethanol extract: 22.1 µg/mL	
Baijiu DG	Polyphenols	DPPH	IC_50_ of purified extract: 34.03 μg/mL IC_50_ of unpurified extract: 16.21 μg/mL	[36]
		ABTS	IC_50_ of unpurified extract: 20.31 μg/mL IC_50_ of unpurified extract: 5.73 μg/mL	
Baijiu DG	Flavonoids	DPPH	62.3% at 79 mg/mL (methanol extract)	[38]
Baijiu DG	Phenols and anthocyanins	DPPH	Above 70% at 1 mg/mL (red yeast rice DG) Above 40% at 1 mg/mL (sorghum DG)	[56]
Dried DSG	Phenols	ABTS	0.32 and 0.44 mg TE/g of aqueous extract 0.54~0.57 mg TE/g of ethanolic extract	[57]
Baijiu DG	Polyphenols	FRAP	2.60~2.61 mg TE/mL of free polyphenols 0.19~0.22 mg TE/mL of bound polyphenols	[58]
		ABTS	7.11~7.38 mg TE/mL of free polyphenols 2.22~3.33 mg TE/mL of bound polyphenols	
		DPPH	5.46~5.71 mg TE/mL of free polyphenols 0.79~1.85 mg TE/mL of bound polyphenols	
Baijiu DG	Polyphenols	DPPH	64.37% (70% acetone vinasse extract)	[59]
		FRAP	4.63 mM FE(II)/g of 70% acetone vinasse extract	

BSG: brewer’s spent grain; DDGS: distiller’s dried grains with solubles; DSG: distiller’s spent grain; DPPH: 1,1-diphenyl-2-picrylhydrazyl; ABTS: 3-ethylbenzothiazoline-6-sulfonic acid; SOD: superoxide dismutase; ORAC: oxygen radical absorbance capacity; FRAP: ferric reducing antioxidant power; TE: Trolox equivalents; PLE: pressurised liquids; UAE: ultrasound.

A significant reduction in IL-6 production was observed in concanavalin A (ConA)-stimulated Jurkat T cells; however, none of the digested milk samples had immunomodulatory effects on RAW264.7 murine macrophages. Cian et al. [60] studied BSG hydrolysates that had anti-inflammatory effects associated with the reduction in TNF and IL-10 production in rat macrophages. The major involvement of the TLR4-NF-*κ*B/MAPK signalling pathway suggested that BSG hydrolysates regulate the immune response, an effect partly maintained after gastrointestinal digestion.

### 3.3. Antihypertensive Activity

ACE is a key enzyme in the classical circulatory renin–angiotensin system and plays an important role in regulating human blood pressure [61]. ACE inhibitors are widely used as natural medicines for the prevention and management of hypertension. Natural ACE inhibitors are believed to have improved safety profiles and fewer side effects [62]. Wei et al. [27] were the first to develop a targeted proteomics approach for peptide identification and identified 22 peptides from DSGs with ACE inhibitory activity. Of these identified peptides, PR had the highest content in the water-soluble extract of dried DSGs (92.14 μg/g dry weight) and acted as a competitive ACE inhibitor (IC_50_ = 50 μM).

Two peptides were isolated and identified from Chinese fermented grains [62], and their ACE inhibitory activities were studied. The results showed that YGD had higher ACE inhibitory activity (IC_50_ = 5.21 μM) than VNP (IC_50_ = 38.02 μM). He et al. [63] isolated two antihypertensive peptides from a rice wine lee extract. LIIPEH and LIIPQH showed strong ACE inhibitory activity, with IC_50_ values of 60.49 and 120.10 μg/mL, respectively. They were further classified as mixed-type ACE inhibitors and were stable against in vitro gastrointestinal digestion.

López-Fernández-Sobrino et al. [64,65,66] investigated the antihypertensive properties of wine lees in spontaneously hypertensive rats (SHR) and discovered that the blood pressure-lowering effect was related to higher amounts of flavonols and anthocyanins, possibly inhibiting ACE. Bravo et al. [67] demonstrated for the first time that a wine lees hydrolysate was a source of ACE inhibitory and antihypertensive peptides with great potential as functional ingredients. Six novel antihypertensive peptides with strong ACE inhibitory activity were identified using nano-HPLC (Orbitrap) MS/MS. Of these, TVTNPARIA, PAGELHP, and LDSPSEGRAPG had ACE inhibitory activity lower than 20 µM.

Ribeiro-Oliveira et al. [68] used simulated oral administration to evaluate the ACE inhibitory potential of a BSG protein hydrolysate. An amount of 0.86 mg/mL of the BSG protein hydrolysate caused a greater reduction in ACE activity than captopril (1 µM), indicating this BSG hydrolysate as a promising natural product to manage hypertension. Garzón et al. [69] used in silico predictions to identify 16 peptides from the BSG hydrolysate that could act as antihypertensive bioactive compounds with multifunctional potential. Cermeño et al. [70] used SHR to evaluate the potent antihypertensive effects of BSG hydrolysates. An alcalase-flavourzyme BSG hydrolysate reduced systolic blood pressure, illustrating the potential of food protein hydrolysates to alleviate hypertension. The hydrolysate was fractionated and subjected to peptide identification, and the IPLQP and LPLQP peptides demonstrated the highest ACE inhibitory activities (IC_50_ = 3.10 and 3.17 µM, respectively). Thus, the antihypertensive effect of this BSG hydrolysate may contribute to ACE inhibition.

### 3.4. Antihyperglycaemic Activity

Inhibition of dipeptidyl peptidase IV (DPP-IV) is a strategy to improve glycaemic regulation in patients with type 2 diabetes. This highly specific serine protease is responsible for the inactivation of glucagon-like peptide-1 (GLP-1) and glucose inhibitory polypeptides (GIP), potentially leading to high serum glucose levels [71]. Hatanaka et al. [72] evaluated the potential of antidiabetic peptides from sake lees subjected to enzymatic digestion using a protease (Denazyme AP). They exhibited DPP-IV inhibitory activity with an IC_50_ value of 27.55 mg/mL. Connolly et al. [73] reported that BSG hydrolysates prepared using proteolytic enzymes were subjected to SGID, which increased the DPP-IV inhibitory activity of the CorPPFla hydrolysate (35 to 87%). In another report, the in vitro DPP-IV inhibitory activity of a BSG protein-enriched isolate, the Alcalase™ hydrolysate (AlcH), was determined. Exposure of AlcH to SGID increased its DPP-IV inhibitory activity, particularly after the intestinal phase of digestion. This was the first study to report on the DPP-IV inhibitory effect of the novel peptides ILLPGAQDGL (IC_50_ = 145.5 μM) and ILDL (IC_50_ = 112.1 μM) [74]. Cermeño et al. [70] analysed the blood pressure-reducing properties of BSG hydrolysates in an SHR model. The hydrolysates were then fractionated, and the peptide IPVP was identified as having the highest DPP-IV inhibitory activity (IC_50_ = 38.67 µM). Further studies exploring the anti-hyperglycaemic potential of BSG peptides in vivo are needed to demonstrate their effects and to develop foods or supplements with benefits against diabetes.

### 3.5. Other Biological Activities

Kawakami et al. [75] reported the hepatoprotective effect of a sake lees hydrolysate against acetaminophen-induced hepatotoxicity through antioxidant effects mediated by hemeoxygenase-1 and γ-glutamylcysteine synthetase expression. Jeon et al. [76] found that trilinolein and triolein isolated from sake lees inhibited tyrosinase activity noncompetitively (IC_50_ = 8.4 and 30 μM, respectively), suggesting great potential for application in the cosmetic field. After optimising the microwave-assisted extraction process, red wine lee extracts from Attica displayed high antimicrobial activity against both Gram-positive and Gram-negative strains [77].

Cian et al. [78] reported that SGID reduced the antithrombotic activity of BSG hydrolysates. The hydrolysates contained medium- and low-molecular-weight (MW) peptides, and hydrophobic amino acids represented the most abundant residues. Low-MW peptides generated after gastrointestinal digestion were responsible for the increased antithrombotic activity of the dialysates of BSG peptides after SGID. All samples of BSG peptides after SGID showed delayed thrombin and activated partial thromboplastin times. Thus, the BSG peptides exerted their antithrombotic activity by inhibiting intrinsic and common pathways of blood coagulation.

## 4. Conclusions and Prospects

This review presents a comprehensive overview of the chemical composition and biological activity of DGs, providing valuable information for the rational utilisation of DG resources. DGs are a good source of phenols, phytosterols, and fatty acids; however, only carbohydrates and proteins in DGs are currently being put to good use in the livestock industry. As reviewed above, other DG constituents showed biological activity and have a great potential for application in human health. Polyphenols are relatively abundant and the most valuable constituents of DGs for further development.

Although DGs have shown various potential health benefits, such as antioxidant, anti-inflammatory, and antihypertensive effects, product development based on these functions is still in its infancy. Challenges appear in diverse areas. For example, dietary fibre from DGs can be added to food products such as bread and biscuits to increase their nutritive value, but the particular smell and taste of DGs must be addressed first. In addition, the bioactive composition of DGs is not fully understood and requires further exploration to lay a solid theoretical foundation for the development of high-value-added products.

## Data Availability

Information is available in the public domain.
